# Association between Virulence Factors and *TRAF1/4-1BB/Bcl-xL* Expression in Gastric Mucosa Infected with *Helicobacter pylori*

**DOI:** 10.1155/2015/648479

**Published:** 2015-02-08

**Authors:** Fen Wang, Xiang Wu, Zhiying Liu, Guangkui Bu, Xiayu Li, Nanfang Qu, Jin Peng, Canxia Xu, Shourong Shen, Yi Yuan

**Affiliations:** ^1^Department of Gastroenterology, The Third Xiangya Hospital of Central South University, Changsha 410013, China; ^2^Department of Parasitology, Xiangya School of Medicine, Central South University, Changsha 410013, China; ^3^Department of Neurology, The Third Xiangya Hospital of Central South University, Changsha 410013, China

## Abstract

*Objective*. *CagA+/vacAs1+/vacAm1+ Helicobacter pylori* upregulates the expression of tumor necrosis factor receptor–associated factor 1 (*TRAF1*), tumor necrosis factor receptor superfamily member 9 (*4-1BB*), and B-cell lymphoma-extra large (*Bcl-xL*) in human gastric epithelial cells. We investigated the correlation between *cagA/vacAs1/vacAm1* and *TRAF1/4-1BB/Bcl-xL* expression in gastric mucosal tissue of patients with gastric disorders. *Methods*. We collected gastric mucosa samples from 35 chronic, nonatrophic gastritis (CG) patients, 41 atrophic gastritis patients, 44 intestinal metaplasia with atypical hyperplasia (IM) patients, and 28 gastric carcinoma (Ca) patients. The expression of  *TRAF1*, *4-1BB*, and *Bcl-xL* was determined using western blotting. The expression of *cagA, vacAs1*, and *vacAm1* in *H. pylori* was examined with polymerase chain reaction. *Results*. The expression of *TRAF1*, *4-1BB*, and *Bcl-xL* was significantly upregulated in IM and Ca patients (*P* < 0.05 compared with CG). There were more cases of *cagA+/vacAs1+/vacAm1+ H. pylori* infection in samples with elevated *TRAF1*, *4-1BB*, or *Bcl-xL* expression (*P* < 0.05). Additionally, there were a remarkably large number of samples with upregulated *TRAF1/4-1BB/Bcl-xL* expression in cases of *cagA+/vacAs1+/vacAm1+ H. pylori* infection (44 cases, 67.7%; *P* < 0.05). *Conclusions*. The pathogenesis of IM and Ca may be promoted by *cagA+/vacAs1+/vacAm1+ H. pylori*, possibly via upregulated *TRAF1*, *4-1BB*, and *Bcl-xL* in gastric mucosal tissue.

## 1. Introduction

*Helicobacter pylori*, a gram-negative bacterium present in nearly 50% of the global population [1, 2], is one of the main causes of peptic ulcer disease [3]. In addition,* H. pylori* infection is related to gastric carcinoma, possibly inducing chronic gastritis, and progresses to the premalignant stages of atrophic gastritis, intestinal metaplasia, and, eventually, gastric carcinoma [4–6]. However, knowledge of the pathogenesis of* H. pylori* infection and gastric diseases is incomplete.

*H. pylori* strains that express virulence genes such as* cagA* and* vacA* are linked to increased risk of gastric cancer [7]. Previously, we performed a comparative genomic study of gastric epithelial cells cocultured with* H. pylori* and found that the expression of tumor necrosis factor receptor–associated factor 1 (*TRAF1*),* 4-1BB* (also known as* CD137*), and B-cell lymphoma-extra large (*Bcl-xL*) was significantly upregulated in human gastric epithelial GES-1 cells infected with* H. pylori* expressing the virulence genotype* cagA+/vacAs1+/vacAm1+* [8]. TRAF1 expression in human gastric mucosa is related to the* H. pylori* virulence genotype* cagA+/vacAs1+/vacAm1+ *[9], whereas the correlation of* 4-1BB* and* Bcl-xL *gene expression with* cagA, vacAs1, *and* vacAm1* toxicity at different stages of gastric disease have not been studied.

In the present study, we investigated* TRAF1, 4-1BB*, and* Bcl-xL* expression in gastric mucosa derived from* H. pylori*–infected patients with chronic nonatrophic gastritis (CG), atrophic gastritis (AG), intestinal metaplasia with atypical hyperplasia (IM), and gastric carcinoma (Ca). We also determined the association between* H. pylori* virulence factors (*cagA, vacAs1, vacAm1*) and* TRAF1/4-1BB/Bcl-xL* expression at the different stages of gastric disease. Our findings may provide valuable insight into the pathogenesis and progression of chronic gastritis into gastric carcinoma.

## 2. Materials and Methods

### 2.1. Reagents

DNA marker and 2x Tag Master Mix were purchased from Beijing CoWin Biotech (Beijing, China). Polyvinyl difluoride (PVDF) membrane was obtained from Millipore (Billerica, MA, USA). PageRuler Prestained Protein Ladder was obtained from Thermo Fisher Scientific (Waltham, MA, USA). Primary antibodies against* TRAF1 *and* Bcl-xL* were purchased from Cell Signaling Technology (Beverly, MA, USA). Mouse monoclonal anti–glyceraldehyde-3-phosphate dehydrogenase (GAPDH) antibody was obtained from Millipore. Anti–*4-1BB* antibody was obtained from Abcam (Cambridge, MA, USA). Goat anti-rabbit and anti-mouse immunoglobulin G (IgG) horseradish peroxidase- (HRP-) conjugated secondary antibodies were obtained from Santa Cruz Biotechnology (Santa Cruz, CA, USA). Unless otherwise stated, all other reagents were provided by the Central South University Cancer Research Institute, Changsha, China.

### 2.2. Sample Collection

In total, 148 gastric antrum samples were collected from patients with different gastric mucosal diseases at the Gastroscope Room, The Third Xiangya Hospital of Central South University, Changsha, China, from January 2013 to December 2013.* H. pylori* infection was determined via rapid urease test, C13 urea breath test, and histological examination. All recruited subjects were* H. pylori* positive;* H. pylori*–positive results were confirmed by at least two of the above tests. Among the patients, 35 had CG, 41 had AG, 44 had IM, and 28 had Ca. Subjects were recruited after they had signed an informed consent. Ethical approval for the study was granted by The Third Xiangya Hospital of Central South University.

### 2.3. Western Blotting

Total protein was extracted from the tissue, and protein concentrations were measured using a bicinchoninic acid protein assay kit according to the manufacturer's instructions (Beijing CoWin Biotech). Equal amounts of protein extracts were separated using sodium dodecyl sulfate–polyacrylamide gel electrophoresis and transferred to PVDF membranes. The PVDF membranes were blocked before they were incubated with primary antibodies at 4°C overnight. The primary antibodies used were anti-*TRAF1* (1 : 500), anti–*4-1BB* (1 : 500), anti–*Bcl-xL* (1 : 1000), and anti-GAPDH (1 : 5000). After washing, the membranes were incubated with goat anti-rabbit or anti-mouse IgG HRP-labeled secondary antibodies (1 : 5000). Immunobands were visualized using an enhanced chemiluminescence kit according to the manufacturer's instructions (Beijing CoWin Biotech). The densitometric values of the immunobands were used for statistical analysis. GAPDH was used as the internal control. Data were quantified from at least three independent experiments.

### 2.4. Polymerase Chain Reaction

Total DNA was extracted from* H. pylori*–infected gastric mucosal tissue using a General AllGen Kit for genomic DNA extraction according to the manufacturer's instructions (Beijing CoWin Biotech). The purity and concentration of the extracted DNA was measured using an ultraviolet spectrometer with an optical density (OD) 260/OD280 ratio. The primers for the* H. pylori* virulence factors were designed as described previously [10]. The forward and reverse primers and PCR product length are listed in Table 1. The PCR conditions were as follows: initial denaturation at 95°C for 5 min; denaturation at 95°C for 30 s; annealing at different temperatures (57°C (*cagA*), 52°C (*vacAs1*), 56°C (*vacAm1*)) for 30 s; and extension at 72°C for 40 s for a total of 39-40 cycles, followed by a final extension at 72°C for 7 min [11].

### 2.5. Statistical Analysis

Data were analyzed using SPSS 17.0 software (IBM, New York, NY, USA). Measurement data are presented as the means ± standard deviation. Comparison among measurement data in different groups was conducted with diverse mean comparison of analysis of variance. A chi-square test was used for comparing enumeration data from different groups. Data correlation was analyzed using Spearman's analysis. *P* < 0.05 was considered significantly different.

## 3. Results

### 3.1. *TRAF1*,* 4-1BB*, and* Bcl-xL* Expression in Gastric Mucosal Tissue

**Figure 1:**
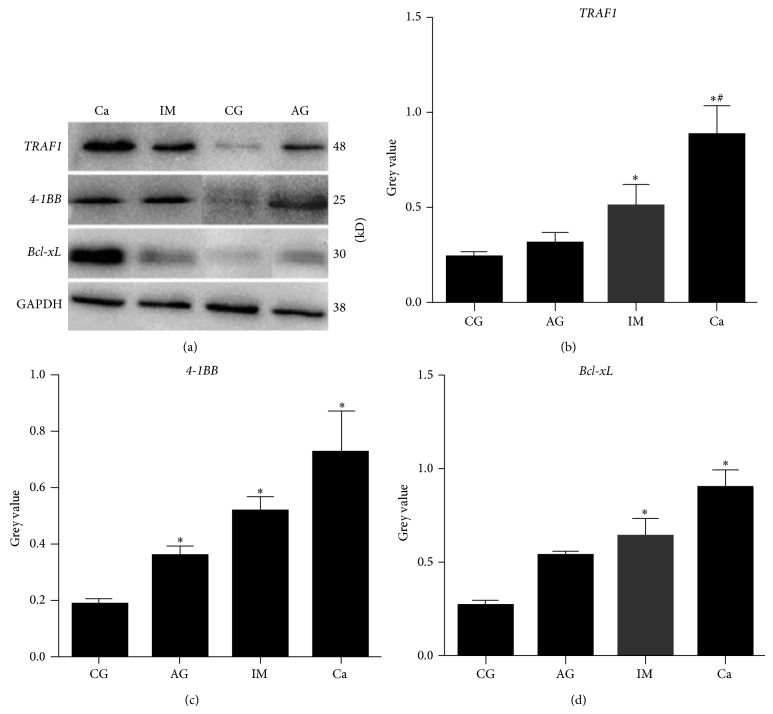
Western blotting examination of* TRAF1, 4-1BB, *and* Bcl-xL* protein expression in gastric mucosal tissue from subjects with CG, AG, IM, or Ca. (a) Representative western blots. Relative expression of (b)* TRAF1*, (c)* 4-1BB,* and (d)* Bcl-xL* was quantified against GAPDH. Data were quantified from three independent experiments. ^*^ *P* < 0.05 compared with CG; ^#^ *P* < 0.05 compared with AG.

First, we examined* TRAF1, 4-1BB, *and* Bcl-xL* protein expression in gastric mucosal tissue from subjects with CG, AG, IM, and Ca using western blotting. As seen in Figure 1,* TRAF1, 4-1BB, *and* Bcl-xL* levels were significantly upregulated in IM and Ca gastric mucosa as compared with CG gastric mucosa (*P* < 0.05). The expression of* 4-1BB* was also elevated in AG gastric mucosa compared to CG gastric mucosa (*P* < 0.05). Moreover, compared to AG gastric mucosa, there was an obvious increase in* TRAF1* protein expression in Ca gastric mucosa (*P* < 0.05). These findings show that the expression of* TRAF1, 4-1BB, and Bcl-xL* was upregulated, especially in the gastric mucosa of IM and Ca patients in comparison to the other groups. Spearman's analysis indicated that the expression of* TRAF1, 4-1BB, *and* Bcl-xL *was positively correlated. The* TRAF1 *and* 4-1BB, TRAF1 *and* Bcl-xL, *and* 4-1BB *and* Bcl-xL* correlation coefficient were 0.678, 0.702, and 0.694, respectively (*P* < 0.05 for all three groups).

### 3.2. Analysis of Virulence Factors in* H. pylori* Extracted from Gastric Mucosa

**Figure 2:**
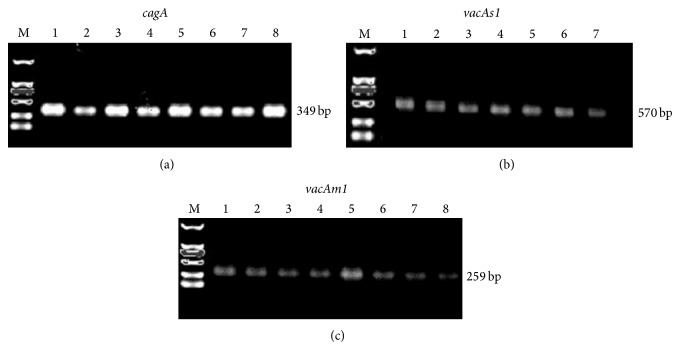
PCR amplification of (a)* cagA*, (b)* vacAs1*, and (c)* vacAm1* expression in* H. pylori* extracted from gastric mucosal tissue. Lane M, DNA marker; numbered lanes, samples with positive expression of the target genes. Base pair value indicates fragment of interest.

We analyzed* H. pylori* virulence factors expression,* that is, cagA, vacAs1, *and* vacAm1*, in* H. pylori* extracted from the different gastric mucosal tissues. Representative positive expression of the virulence factors is presented in Figure 2. To clarify the association between the* H. pylori* virulence factors and* TRAF1, 4-1BB, *and* Bcl-xL* expression, the number of samples positive or negative for* cagA, vacAs1, *and* vacAm1 *was stratified according to* TRAF1, 4-1BB,* and* Bcl-xL* expression (Table 2). According to the results of western blot, the gastric cancer and intestinal metaplasia with atypical hyperplasia groups were classified as the* TRAF1, 4-1BB*, and* Bcl-xL* high expression group, while the gastritis and atrophic gastritis groups were classified as the* TRAF1, 4-1BB*, and* Bcl-xL* low expression group. The number and percentage of* cagA-, vacAs1-, *and* vacAm1-*positive cases were greatly upregulated in samples with high* TRAF1, 4-1BB, *or* Bcl-xL* expression (*P* < 0.05 compared with cases negative for* cagA, vacAs1, vacAm1*). In addition, the number of* cagA+/vacAs1+/vacAm1+* cases in samples with elevated* TRAF1, 4-1BB, *or* Bcl-xL *expression was increased dramatically (*P* < 0.05 compared with unaltered* TRAF1, 4-1BB, *or* Bcl-xL *expression).

### 3.3. Correlation between* cagA/vacAs1/vacAm1* and* TRAF1/4-1BB/Bcl-xL* Expression

To explore the potential association between* cagA/vacAs1/vacAm1* and* TRAF1/4-1BB/Bcl-xL* expression, we analyzed the number and percentage of samples with positive or negative* cagA/vacAs1/vacAm1* expression and differential* TRAF1/4-1BB/Bcl-xL *expression. A remarkably large number of samples with upregulated* TRAF1/4-1BB/Bcl-xL* expression were* cagA+/vacAs1+/vacAm1+* (44 cases, 67.7%; *P* < 0.05; Table 3). From these findings, we believe that* cagA+/vacAs1+/vacAm1+ H. pylori* may promote the IM and Ca pathogenesis, possibly by upregulating* TRAF1, 4-1BB, *and* Bcl-xL* expression in the gastric mucosal tissue.

## 4. Discussion

Previously, we found that* TRAF1, 4-1BB, *and* Bcl-xL* were significantly upregulated in GES-1 cells infected with* H. pylori* expressing the virulence genotype* cagA+/vacAs1+/vacAm1+* [8]. Here, we show that expression of* TRAF1, 4-1BB, *and* Bcl-xL* was significantly upregulated in IM and Ca patients compared with CG patients. Moreover, compared to samples in which* TRAF1, 4-1BB, *or* Bcl-xL* expression was unaltered, there were more* cagA+/vacAs1+/vacAm1+ H. pylori*–infected cases in samples with elevated* TRAF1, 4-1BB*, or* Bcl-xL* expression.

*TRAF1* belongs to a group of structurally similar adapter proteins (TRAFs), but differs from other* TRAFs* because it lacks the conserved N-terminal RING domain found in other TRAF family proteins [12].* TRAF1* plays a critical role in regulating apoptosis by indirectly modulating the transcription factor nuclear factor-*κ*B inducible gene expression [12]. In a previous study, we found that* TRAF1* was upregulated in several gastric cancer cell lines, including BGC823, SGC7901, and MGC803 [13]. Moreover,* TRAF1* expression in human gastric mucosa is related to the* H. pylori* virulence genotype* cagA+/vacAs1+/vacAm1+* [9]. In accordance with these findings, we demonstrate here that* TRAF1* was highly expressed in the gastric mucosa of IM and Ca patients and that* TRAF1* upregulation correlated positively with the* H. pylori cagA+/vacAs1+/vacAm1+* virulence genotype.

*Bcl-xL and 4-1BB* are two key regulators in the* TRAF1* signaling pathway.* TRAF1* has a prosurvival effect in CD8 T cells via the* 4-1BB*–mediated upregulation of* Bcl-xL* [14].* Bcl-xL*, an antiapoptotic member of the Bcl-2 family, is involved in modulating the angiogenic phenotype of human tumor cells [15, 16]. Via cross-talk with P-glycoprotein,* Bcl-xL* acts as an antiapoptotic factor in* H. pylori*–related gastric carcinogenesis [17]. Hence, we investigated* 4-1BB *and* Bcl-xL* expression in the gastric mucosa from different gastric diseases.* 4-1BB* expression was greatly upregulated in AG, IM, and Ca gastric mucosa, and* Bcl-xL* levels were increased, especially in IM and Ca gastric mucosa. Consistent with our findings, Yang et al. reported that* Bcl-xL* expression was relatively low in CG and AG patients, but was markedly increased in Ca patients [18]. As* TRAF1, 4-1BB,* and* Bcl-xL* are correlated positively,* TRAF1* may trigger* 4-1BB*–mediated* Bcl-xL* activity.* TRAF1–4-1BB–Bcl-xL* signaling pathway upregulation may play a critical role in the development gastritis into gastric cancer.

A significantly high prevalence of East Asian* cagA*-positive* H. pylori* infection has been reported in gastric cancer patients (84.6%), suggesting that* cagA*-positive* H. pylori* infection and gastric cancer are closely associated [19]. Furthermore, it has been suggested that the* vacAs1+/vacAm1+* genotype is associated with gastric cancer [20]. Importantly, a high proportion of subjects with upregulated* TRAF1, 4-1BB, *and* Bcl-xL *expression in the present study were infected with* cagA+/vacAs1+/vacAm1+ H. pylori*. In contrast, Matsumoto et al. showed that* H. pylori vacA* reduced* Bcl-xL* expression in gastric adenocarcinoma cell lines and led to apoptosis [21]. This discrepancy might be due to the differences between* in vitro* and* in vivo* conditions. Additionally, we cannot discount the possibility that differing virulence genotypes (*cagA+/vacAs1+/vacAm1+, vacA+*) may have different effects. Future studies should continue to investigate the effects of the* cagA+/vacAs1+/vacAm1+* virulence genotype on* TRAF1, 4-1BB, *and* Bcl-xL* expression in cultured gastric cancer cells.

In summary, our study implies that* cagA+/vacAs1+/vacAm1+ H. pylori *infection might promote gastritis progression to gastric cancer, possibly by upregulating* TRAF1, 4-1BB, *and* Bcl-xL *expression in gastric mucosal tissue. It is possible that* cagA+/vacAs1+/vacAm1+ H. pylori* upregulates* TRAF1* activation, which triggers* 4-1BB*–mediated* Bcl-xL* activation, thereby exerting an antiapoptotic effect and contributing to the pathogenesis of gastric cancer. Nevertheless, the underlying mechanism involved in this process requires further clarification.

## Figures and Tables

**Table 1:** Primers and PCR conditions.

Gene	Primers (forward, reverse)	Annealing temperature (°C)	Amplified product length (bp)
*cagA *	5′-GATAACAGGCAAGCTTTTGAGG-3′	57	349
	5′-CTGCAAAAGATTGTTTGGCAGA-3′		

*vacAs1 *	5′-ATGGAAATACAACAAACACAC-3′	52	259
	5′-CTGCTTGAATGCGCCAAAC-3′		

*vacAm1 *	5′-CAATCTGTCCAATCAAGCGAG-3′	56	570
	5′-GCGTCTAAATAATTCCAAGG-3′		

**Table 2:** Samples positive or negative for *cagA, vacAs1,* or *vacAm1* stratified according to* TRAF1, 4-1BB, *and *Bcl-xL* expression.

Group	*TRAF1 *		*4-1BB *		*Bcl-xL *	
	↑	—	↑	—	↑	—
*cagA+/vacAs1+/vacAm1+ *	50 (64.9)^*^	15 (21.1)	50 (66.7)^*^	15 (20.5)	47 (59.4)^*^	18 (26.1)
*cagA+/vacAs1+/vacAm1− *	6 (7.8)	10 (14.1)	7 (9.3)	9 (12.3)	4 (5.1)	12 (17.4)
*cagA+/vacAs1−/vacAm1+ *	3 (3.9)	12 (16.9)	3 (4.0)	12 (16.4)	7 (8.9)	8 (11.6)
*cagA+/vacAs1−/vacAm1− *	3 (3.9)	10 (14.1)	3 (4.0)	10 (13.7)	3 (3.8)	10 (14.5)
*cagA−/vacAs1+/vacAm1+ *	3 (3.9)	6 (8.5)	5 (6.7)	4 (5.5)	5 (6.3)	4 (5.8)
*cagA−/vacAs1+/vacAm1− *	5 (6.5)	8 (11.3)	1 (1.3)	12 (16.4)	3 (3.8)	10 (14.5)
*cagA−/vacAs1−/vacAm1+ *	2 (2.6)	5 (7.0)	3 (4.0)	4 (5.5)	4 (5.1)	3 (4.3)
*cagA−/vacAs1−/vacAm1− *	5 (6.5)	5 (7.0)	3 (4.0)	7 (9.6)	6 (7.6)	4 (5.8)
*cagA+ *	62 (80.5)	47 (66.2)	63 (84.0)	46 (63.0)	61 (77.2)	48 (69.6)
*cagA− *	15 (19.5)	24 (33.8)	12 (16.0)	27 (37.0)	18 (22.8)	21 (30.4)
*vacAs1+ *	64 (83.1)	39 (54.9)	63 (84.0)	40 (54.8)	59 (74.7)	44 (63.8)
*vacAs1− *	13 (16.9)	32 (45.1)	12 (16.0)	33 (45.2)	20 (25.3)	25 (36.2)
*vacAm1+ *	58 (75.3)	38 (53.5)	61 (81.3)	35 (47.9)	63 (79.7)	33 (47.8)
*vacAm1− *	19 (24.7)	33 (46.5)	14 (18.7)	38 (52.1)	16 (20.3)	36 (52.2)

Total	77 (100.0)	71 (100.0)	75 (100.0)	73 (100.0)	79 (100.0)	69 (100.0)

↑, upregulated; —, unaltered. ^*^ *P* < 0.05 compared with unaltered group. Data are presented as the number of cases and percentage (%).

**Table 3:** Correlation between expression of *cagA, vacAs1, *and *vacAm1* and *TRAF1, 4-1BB, *and *Bcl-xL*.

Group	T^+^/4^+^/B^+^	T^+^/4^+^/B^−^	T^+^/4^−^/B^+^	T^+^/4^−^/B^−^	T^−^/4^+^/B^+^	T^−^/4^+^/B^−^	T^−^/4^−^/B^+^	T^−^/4^−^/B^−^	Total
*cagA+/vacAs1+/vacAm1+ *	44 (67.7)^*^	4 (6.2)	1 (1.5)	1 (1.5)	0 (0.0)	2 (3.1)	2 (3.1)	11 (16.9)	65 (100.0)
*cagA+/vacAs1+/vacAm1− *	2 (12.5)	2 (12.5)	0 (0.0)	2 (12.5)	2 (12.5)	1 (6.3)	0 (0.0)	7 (43.8)	16 (100.0)
*cagA+/vacAs1−/vacAm1+ *	1 (6.7)	0 (0.0)	2 (13.3)	0 (0.0)	2 (13.3)	0 (0.0)	2 (13.3)	8 (53.3)	15 (100.0)
*cagA+/vacAs1−/vacAm1− *	1 (7.7)	0 (0.0)	1 (7.7)	1 (7.7)	0 (0.0)	2 (15.4)	1 (7.7)	7 (53.8)	13 (100.0)
*cagA−/vacAs1+/vacAm1+ *	2 (22.2)	0 (0.0)	1 (11.1)	0 (0.0)	2 (22.2)	1 (11.1)	0 (0.0)	3 (33.3)	9 (100.0)
*cagA−/vacAs1+/vacAm1− *	1 (7.7)	0 (0.0)	2 (15.4)	2 (15.4)	0 (0.0)	0 (0.0)	0 (0.0)	8 (61.5)	13 (100.0)
*cagA−/vacAs1−/vacAm1+ *	1 (14.3)	0 (0.0)	0 (0.0)	1 (14.3)	2 (28.6)	0 (0.0)	1 (14.3)	2 (28.6)	7 (100.0)
*cagA−/vacAs1−/vacAm1− *	3 (30.0)	0 (0.0)	1 (10.0)	1 (10.0)	0 (0.0)	0 (0.0)	2 (20.0)	3 (30.0)	10 (100.0)

^*^ *P* < 0.05 compared with other groups. T, *TRAF1*; 4, *4-1BB*; B, *Bcl-xL*. Data are presented as the number of cases and percentage (%).

## References

[1] McColl K. E. L. (2010). Helicobacter pylori infection. *The New England Journal of Medicine*.

[2] Suerbaum S., Michetti P. (2002). *Helicobacter pylori* infection. *The New England Journal of Medicine*.

[3] Malnick S. D., Melzer E., Attali M., Duek G., Yahav J. (2014). *Helicobacter pylori*: friend or foe?. *World Journal of Gastroenterology*.

[4] Correa P. (1992). Human gastric carcinogenesis: a multistep and multifactorial process—first American Cancer Society Award lecture on cancer epidemiology and prevention. *Cancer Research*.

[5] Lu B., Li M. (2014). Helicobacter pylori eradication for preventing gastric cancer. *World Journal of Gastroenterology*.

[6] Wang L., Zou X., Liu Y.-F., Sheng G.-Y. (2013). Association between helicobacter pylori infection and chronic idiopathic neutropenia. *Journal of Huazhong University of Science and Technology—Medical Science*.

[7] Ki M.-R., Hwang M., Kim A.-Y. (2014). Role of vacuolating cytotoxin VacA and cytotoxin-associated antigen CagA of *Helicobacter pylori* in the progression of gastric cancer. *Molecular and Cellular Biochemistry*.

[8] Wang F., Luo L.-D., Pan J.-H. (2012). Comparative genomic study of gastric epithelial cells co-cultured with Helicobacter pylori. *World Journal of Gastroenterology*.

[9] Wang F., Bu G., Feng Q. (2014). The expression level of TRAF1 in human gastric mucosa is related to virulence genotypes of *Helicobacter pylori*. *Scandinavian Journal of Gastroenterology*.

[10] Yamaoka Y., Kodama T., Gutierrez O., Kim J. G., Kashima K., Graham D. Y. (1999). Relationship between Helicobacter pylori iceA, cagA, and vacA status and clinical outcome: studies in four different countries. *Journal of Clinical Microbiology*.

[11] Arévalo-Galvis A., Trespalacios-Rangel A. A., Otero W., Mercado-Reyes M. M., Poutou-Piñales R. A. (2012). Prevalence of cagA, vacA, babA2 and iceA genes in *H. pylori* strains isolated from Colombian patients with functional dyspepsia. *Polish Journal of Microbiology*.

[12] Leo E., Deveraux Q. L., Buchholtz C. (2001). TRAF1 is a substrate of caspases activated during tumor necrosis factor receptor-alpha-induced apoptosis. *The Journal of Biological Chemistry*.

[13] Wang F., Yang Y., Feng Q. (2012). Construction of RNAi targeting TRAF1 gene and effect of TRAF1 on gastric cancer cells. *Zhong Nan Da Xue Xue Bao Yi Xue Ban*.

[14] Sabbagh L., Pulle G., Liu Y., Tsitsikov E. N., Watts T. H. (2008). ERK-dependent Bim modulation downstream of the 4-1BB-TRAF1 signaling axis is a critical mediator of CD8 T cell survival in vivo. *Journal of Immunology*.

[15] Giorgini S., Trisciuoglio D., Gabellini C. (2007). Modulation of bcl-xL in tumor cells regulates angiogenesis through CXCL8 expression. *Molecular Cancer Research*.

[16] Adams J. M., Cory S. (2007). The Bcl-2 apoptotic switch in cancer development and therapy. *Oncogene*.

[17] Rocco A., Compare D., Liguori E. (2012). MDR1-P-glycoprotein behaves as an oncofetal protein that promotes cell survival in gastric cancer cells. *Laboratory Investigation*.

[18] Yang L., Levi E., Zhu S., Du J., Majumdar A. P. N. (2013). Cancer stem cells biomarkers in gastric carcinogenesis. *Journal of Gastrointestinal Cancer*.

[19] Satomi S., Yamakawa A., Matsunaga S. (2006). Relationship between the diversity of the cagA gene of *Helicobacter pylori* and gastric cancer in Okinawa, Japan. *Journal of Gastroenterology*.

[20] Kidd M., Lastovica A. J., Atherton J. C., Louw J. A. (1999). Heterogeneity in the *Helicobacter pylorivacA* and *cagA* genes: association with gastroduodenal disease in South Africa?. *Gut*.

[21] Matsumoto A., Isomoto H., Nakayama M. (2011). Helicobacter pylori VacA reduces the cellular expression of STAT3 and pro-survival Bcl-2 family proteins, Bcl-2 and Bcl-X L, leading to apoptosis in gastric epithelial cells. *Digestive Diseases and Sciences*.

